# Occult HBV infection in Chinese blood donors: role of N-glycosylation mutations and amino acid substitutions in S protein transmembrane domains

**DOI:** 10.1080/22221751.2019.1663130

**Published:** 2019-09-13

**Authors:** Lu Zhang, Le Chang, Syria Laperche, Huimin Ji, Junpeng Zhao, Xinyi Jiang, Lunan Wang, Daniel Candotti

**Affiliations:** a National Center for Clinical Laboratories, Beijing Hospital, National Center of Gerontology, Beijing, People’s Republic of China; b Beijing Engineering Research Center of Laboratory Medicine, Beijing Hospital, Beijing, People’s Republic of China; c Graduate School, Peking Union Medical College, Chinese Academy of Medical Sciences, Beijing, People’s Republic of China; d National Institute of Blood Transfusion, DATS, CNR RIT, Paris, France

**Keywords:** Hepatitis B virus, occult infection, N-linked glycosylation, transmembrane domain, blood donors, HBsAg

## Abstract

Occult hepatitis B virus infection (OBI) is a low-level asymptomatic phase of HBV infection. Evidence of OBI clinical relevance is emerging but the mechanisms of its occurrence remain unclear. In this study, the molecular characteristics of 97 confirmed OBI from Chinese blood donors were analyzed and relevant mutations were identified. Recombinant HBsAg bearing these mutations were expressed in vitro and the antigenicity and HBsAg secretion properties were analyzed. Results showed that 45 (46.4%) genotype B, 50 (51.5%) genotype C, and 2 (2.1%) genotype D sequences were identified. Two groups of mutations in the S gene were significantly associated with OBI. The first group included mutations creating new N-linked glycosylation sites at positions s116, s123, s130, and s131 + s133 or removing the existing one at s146. Mutations TCT123-125NCT/NFT were associated with reduced antigenicity, while TST116-118NST, GTS130-132NTS, and TSM131-133NSS/NYT/NST were associated with varying levels of impaired HBsAg secretion. N146 mutations had no effect on HBsAg production pattern. The second group included substitutions within the S transmembrane domains TMD1-3. Only mutations C85R, L87R, L88R, and C90R within TMD2 were associated with defective HBsAg production. These mutations appear to be rare and mostly strain specific but they may contribute to the multifactorial occurrence of OBI.

## Introduction

The implementation of nucleic acid testing for hepatitis B virus (HBV) DNA has revealed a relatively large number of apparently “healthy” blood donors infected with so-called occult HBV infection/carriage which characterized with undetectable HBsAg. Occult HBV infection (OBI) is defined as the presence of replication competent HBV DNA in the liver (i.e. episomal HBV covalently closed circular DNA) and/or HBV DNA in the blood (usually at levels <200 IU/mL) of individuals who test negative for HBV surface antigen (HBsAg) with currently available assays, in the presence or absence of hepatitis B core antibodies (anti-HBc) and/or hepatitis B surface antibodies (anti-HBs) [1]. OBI has been reported worldwide, although its prevalence varies between different geographic areas and populations according to HBV endemicity and HBV genotypes [2]. The clinical outcomes of OBI remain unclear but it has been associated with the transmission of HBV infection by transfusion and liver transplantation, viral reactivation in immunosuppressed carriers, and mild continuous liver necro-inflammation that may favour progression to cirrhosis and eventually constitute an important risk factor for hepatocellular carcinoma development since the virus appears to maintain its tumorigenic properties [3].

Detection of the full-length viral genome associated with infectious mature viral particle in the plasma of most OBI carriers indicates a sustained but extremely low viral replication imperfectly controlled by the host immune system and/or related to viral variants [1,4]. The molecular mechanisms underlying OBI are not completely understood but appear to be multifactorial and involve viral, host, and epigenetic factors separately or simultaneously [1]. Nevertheless, OBI has been frequently associated with high levels of amino acid (aa) substitutions, particularly within the major hydrophilic region (MHR) of the S protein [5–8]. Some of these substitutions are related to HBsAg detection failure by affecting antigenicity and detection by commercial assays and/or co-translational maturation of S protein and HBsAg secretion [9–11].

The HBV envelope gene encodes three envelope proteins referred as large (L), middle (M) and small (S), which share the S domain at their C termini constituting the HBsAg. All three proteins are produced in the endoplasmic reticulum (ER), self-assembled, and empty subviral particles (SVP) consisting essentially of S proteins and lipids are secreted in a ≤10^4^-excess over virions containing the three proteins S, M, and L at a ratio of approximately 4:1:1 [12]. The envelopes of virions and SVP express the same antigenicity. The S protein contains at least two hydrophobic transmembrane domains (TMDs): N-terminal type I TMD1 (aa 8–22) and a central type II TMD2 (aa 80–98) which anchor the polypeptide chain into the viral membrane. In most existing models, the relatively hydrophobic C-terminal region (HCR, aa 170–226) is assumed to contain two additional transmembrane domains (TMD3 and TMD4), the location of which remain unclear [13]. The loops between TMD1 and TMD2, as well as between TMD2 and the HCR are in the cytosol and ER lumen during synthesis, respectively. The latter is displayed on the surface of secreted particle where it constitutes the MHR bearing the immunodominant a-determinant. The S protein is cotranslationally modified by partial N-linked glycosylation at asparagine residue 146 and by disulfide bridges formation leading to covalently linked S homodimers. However, the number and positions of intra- and inter-molecular disulfide bridges in S dimers remain unknown, although correct disulfide bridges appeared to be fundamental for forming the main HBsAg epitope [14,15]. The N146 glycosylation site is conserved in HBV strains and effective glycosylation has been reported as essential for HBV virion secretion. This site also plays a protective role against neutralizing antibodies, while un-glycosylation appeared essential for infectivity [16–19].

In the present study, the effect of amino acid substitutions in HBV strains infecting OBI carriers that modify the S protein glycosylation pattern or the hydrophobicity of TMD1-3 were examined in vitro.

## Patients and methods

### Samples collection and characterization

Between January 2010 and December 2013, 1,261 HBsAg-/HBV DNA+ blood donor plasmas identified in 29 blood centres located in 19 different provinces in China were shipped to the National Center for Clinical Laboratories (NCCL). At donation time, HBV DNA was tested using either the cobas® TaqScreen MPX Test V2.0 (95% limit of detection: 2.3 IU/mL; Roche Diagnostics, Manheim, Germany) or the PROCLEIX ULTRIO® Plus assay (95% limit of detection: 3.4 IU/mL; Grifols, Barcelona, Spain). Information including age, gender, donor status (repeat or first time), and alanine aminotransferase (ALT) level were available.

These samples were tested further for HBsAg, anti-HBs, HBeAg, anti-HBe, and anti-HBc with ARCHITECT i2000 (Abbott Laboratories, Chicago, IL, USA) in the NCCL. HBV DNA was quantified with Roche cobas AmpliPrep/cobas TaqMan V 2.0 (Roche, Basel, Switzerland). Samples confirmed as HBsAg-/HBV DNA+ (HBV DNA load < 200 IU/mL) which are anti-HBc and/or anti-HBs positive were classified into the OBI group. Samples that were both anti-HBc and anti-HBs negative were excluded in the absence of follow-up because a pre-seroconversion window period could not be ruled out.

### S region amplification and sequencing

HBV DNA was extracted after concentration of viral particles by precipitation with 12% polyethylene glycol from 8 mL plasma, amplified by semi-nested PCR (95% detection limit: 20 IU/mL), and sequenced. Semi-nested PCR was performed by using primers Kp2356sF (5'-TCCGCTAGAGATCTGGTACCATGGCAGGTCCCCTAGAAGAAGAACT-3') and Hd1627R (5'-TCTTATCTAGAAGCTTGCGTTCACGGTGGTCTCCATGC-3') in the first round, Kp2356sF and Hd1599sR (5'-TCTTATCTAGAAGCTTACAGAGGTGAAGCGAAGTGCACACGG-3') in the second round. The 20-μL first round reaction contained 5μL DNA, 0.4 mM dNTP, 0.2 μM each primer, and 0.05 U/μL LA Taq. The second round reaction was performed in 50 μL including 2 μL of first round products, 0.4 mM dNTP, 0.2 μM each primer, and 0.05 U/μL LA Taq. The amplification programme in the first round was 95°C for 4 min, 35 cycles of 94°C for 30 s + 58°C for 30 s + 72°C for 3 min, and 72°C for 10 min. The amplification programme for the second round was identical except that 25 cycles were performed. Purified amplicons were sequenced directly using primers Kp2356sF and Hd1599sR. OBI sequences were aligned with the sequences of 353 genotype B and 162 genotype C sequences from HBsAg+ blood donors used as control groups. Phylogenetic analysis was performed using software Genious, Mega, and online software (https://www.hiv.lanl.gov/content/sequence/VESPA/vespa.html).

### Functional analysis of amino acid substitutions within S protein

Mutations resulting in aa substitutions of potential interest were introduced by site directed mutagenesis (SDM) into the sequence of plasmid P38.II containing a 1.2X genotype C HBV genome under the control of HBV promoters [20], and/or plasmids M86 and M88 containing the genotype C and genotype B small S coding sequences under the control of the human cytomegalovirus (HCMV) immediate early promoter, respectively [10].

Plasmids with mutations and wild-type (wt) controls (500 ng/transfection) were transfected into HuH-7 cells as described previously [10]. Plasmid pSELECT-zeo-SEAP (InvivoGen, San Diego, CA, USA) was co-transfected (50 ng/transfection) for normalization. Experiments were performed at least in triplicate.

Culture supernatants were collected seven days post-transfection. Cells were washed three times with 1X phosphate-buffered saline (PBS) and lysed in 500 μL of 1X PBS, 1% Triton X-100, 10 U/mL DNase I, and complete proteinase inhibitor/EDTA (Roche, Basel, Switzerland). Cell lysates were centrifuged at 13,000 rpm for 15 min, and the supernatants were collected as the cytosolic extracts. HBsAg production was quantified in both extra- (EC) and intra-cellular (IC) fractions by enzyme immunoassay (EIA, Murex HBsAg v3 enzyme immunoassay, DiaSorin, Dartford, UK). A standard curve was generated by testing serial dilutions of a commercial calibrated recombinant HBsAg (Source BioScience, Nottingham, UK). Seap production was tested using the QUANTI-Blue Solution kit (InvivoGen, San Diego, CA, USA). HBsAg production was normalized according to the measured Seap activity. To evaluate HBsAg secretion, the EC/IC ratio of HBsAg production was calculated. All results were expressed as relative value compared to wt plasmids.

### Western blot analysis

Culture supernatants were concentrated by density gradient centrifugation with 1X 30% sucrose TNE at 50, 000 g, and then analyzed by western blotting as previously described [16]. Cytosolic extracts were analyzed in parallel using the same method. PNGase F treatment was performed to confirm the presence of N-glycans.

### Statistical analysis

Statistical analyses were performed using the Student’s t test for quantitative data and the Chi-square test (continuity correction) for qualitative variables. *P* values <0.05 were considered statistically significant.

## Results

### OBI donors identification and characteristics

Among the 1,261 blood donors initially screened as HBsAg-/HBV DNA+, 918 donors (72.8%) were confirmed as OBI carriers. Host and viral markers were available for 906 OBI donors (Table 1). Most OBI donors were male (71.2%), 40.0% were repeat donors, the median age was 41 years (range: 18–60 years), and all had a normal ALT level (≤50 IU/mL). The median HBV DNA load was <12 IU/mL (range: <12–161 IU/mL) with 61.9% of OBI donors showing HBV DNA levels less than 12 IU/mL.

**Table 1.** Host and viral markers in OBI donors stratified according to serological status.

Markers	Total	Anti-HBc + Anti-HBs -	Anti-HBc + Anti-HBs +	Anti-HBc - Anti-HBs +
N (%)	906	634 (70.0%)	215 (23.7%)	57 (6.3%)
Repeat donors (%)	40.0%	38.6%	48.8%	28.1%
Gender (F/M)	261/645	179/455	55/160	27/30
Age (years)				
Median	41	42	43	23
Range	18–60	18–60	18–58	19–59
ALT ≤ 50 IU/ml (%)	100%	100%	100%	100%
HBV DNA load (IU/mL)				
Median	<12	<12	<12	20
Range	<12–161	<12–161	<12–115	<12–146
<12	561 (61.9%)	384 (60.6%)	152 (70.7%)	25 (43.9%)
12–100	335 (37.0%)	245 (38.6%)	62 (28.8%)	28 (49.1%)
100–200	10 (1.1%)	5 (0.8%)	1 (0.5%)	4 (7.0%)
Anti-HBs titer (IU/L)				
Median	32.3	-	29.5	57.4
Range	10.1–>1000	-	10.1–>1000	10.5–834.6
<10	634 (70.0%)	634 (100%)	-	-
10–100	228 (25.2%)	-	188 (87.4%)	40 (70.2%)
100–1000	42 (4.6%)	-	25 (11.6%)	17 (29.8%)
>1000	2 (0.2%)	-	2 (1.0%)	0 (0%)

Additional serological testing identified 634 (70.0%) samples as anti-HBc-only reactive, 215 (23.7%) carrying both anti-HBc and anti-HBs, and 57 (6.3%) as anti-HBs-only reactive (Table 1). Overall, anti-HBs were detectable in 272 (30.0%) OBI donors with a median titer of 32.3 IU/L (range: 10.1–>1000 IU/L). In anti-HBs-only OBI donors, the median antibody titer was 57.4 IU/L (range: 10.5–834.6 IU/L). Anti-HBs-only OBI donors were significantly younger (median age 23 years [range: 19–59]) compared to the other two groups (median 42 [range: 18–60] and 43[range: 18–58] years for anti-HBc+/anti-HBs- and anti-HBc+/anti-HBs+ carriers, respectively) (*P* < 0.01)

### Genetic analysis of the S region of OBI donors

S sequences were obtained from 97/250 (38.8%) OBI samples randomly selected. The host and viral markers in the 97 successfully sequenced OBI samples were similar to those in the 906 OBI samples (Table 2). Sequenced samples included 19 (19.6%) anti-HBc+/anti-HBs+, 69 (71.1%) anti-HBc+/anti-HBs-, and 9 (9.3%) anti-HBc-/anti-HBs+ samples. Phylogenetic analysis identified 45 (46.4%) genotype B, 50 (51.5%) genotype C, and 2 (2.1%) genotype D sequences (Table 2). Additionally, 530 HBsAg+ blood donor samples were sequenced for comparison (353 genotype B [66.6%], 162 genotype C [30.6%], and 15 genotype D [2.8%]).

**Table 2.** Host and viral markers in 97 successfully sequenced OBI samples stratified according to serological status.

Markers	Total	Anti-HBc + Anti-HBs -	Anti-HBc + Anti-HBs +	Anti-HBc - Anti-HBs +
N (%)	97	69 (71.1%)	19 (19.6%)	9 (9.3%)
Repeat donors (%)	45.4%	42.0%	57.9%	44.4%
Gender (F/M)	33/64	14/55	13/6	6/3
Age (years)				
Median	38	38	42	23
Range	19–59	21–55	21–54	19–59
ALT ≤ 50 IU/ml (%)	100%	100%	100%	100%
HBV DNA load (IU/mL)				
Median	<12	<12	<12	53.9
Range	<12–146	<12–103	<12–79.7	<12–146
<12	77 (79.4%)	59 (85.5%)	15 (78.9%)	3 (33.3%)
12–100	16 (16.5%)	9 (13.0%)	4 (21.1%)	3 (33.3%)
100–200	4 (4.1%)	1 (1.5%)	0 (0%)	3 (33.3%)
Anti-HBs titer (IU/L)				
Median	71.7	-	34.9	105.5
Range	11.1–>1000	-	11.1–>1000	13.1–411.4
<10	69 (71.1%)	69 (100%)	-	-
10–100	19 (19.6%)	-	16 (84.2%)	3 (33.3%)
100–1000	8 (8.3%)	-	2 (10.5%)	6 (66.7%)
>1000	1 (1.0%)	-	1 (5.3%)	0 (0%)
HBV genotypes (N)				
B	45 (46.4%)	34 (49.3%)	8 (42.1%)	3 (33.3%)
C	50 (51.5%)	33 (47.8%)	11 (57.9%)	6 (66.7%)
D	2 (2.1%)	2 (2.9%)	0 (0%)	0 (0%)

**Figure 1.**
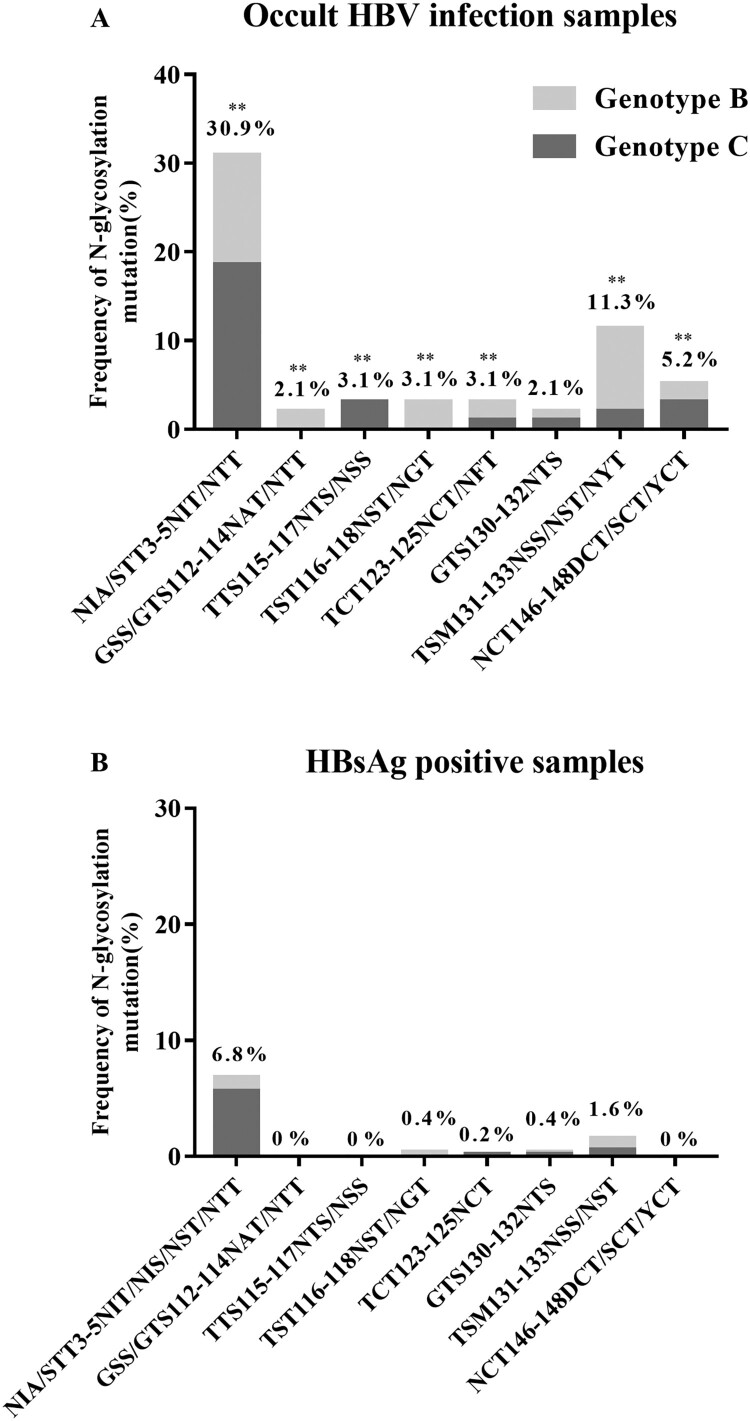
Frequency of N-glycosylation mutations within HBV S sequences from 95 OBIs and 515 HBsAg+ donors. ** *P* < 0.01.

Overall, higher intra-group aa diversity was observed in the S sequences of OBIs compared to non-OBI controls (data not shown). Particularly, mutations removing the N146 glycosylation site (N146D/S/Y) were found in 4.4% (2/45) and 6% (3/50) of OBI genotype B (OBI_B_) and genotype C (OBI_C_) sequences, respectively. No N146 mutation was found in non-OBI controls (Figure 1). Single or double mutations that introduced seven potential additional N-linked glycosylation sites (NXT/S, where X is any aa except proline) at aa positions 3, 112, 115, 116, 123, 130, and 131 were observed more frequently in OBIs than in non-OBIs (44.2% [42/95] vs 9.5% [49/515]; *P* < 0.01). Within MHR, the frequency of N-glycosylation mutations was higher in OBI_B_ than in OBI_C_, and some mutations appeared to be genotype-specific: GSS/GTS112-114NAT/NTT and TST116-118NST/NGT in OBI_B_ and TTS115-117NTS/NSS in OBI_C_ (Figure 1). Detectable levels of anti-HBs (median: 36 IU/L; range: 11–232 IU/L) were observed in 42.9% (18/42) of OBI donors carrying N-linked glycosylation site mutants.

Amino acid variability was further examined in the four putative TMDs of S protein. Amino acid substitutions were significantly more frequent in OBI than in non-OBI TMD sequences (86.3% [82/95] vs 61.9% [319/515]; *P* < 0.01). Because TMDs are alpha helices embedded in a highly hydrophobic environment, we focus on mutations substituting a hydrophobic residue with a hydrophilic one (Table 3). The results showed that hydrophilic substitutions were more frequent in all four TMDs in OBIs than in non-OBIs (64.2% [61/95] versus 24.7% [127/515]; *P* < 0.01).

**Table 3.** Frequency of HBV strains from HBsAg+ and OBI blood donors showing hydrophilic amino acid substitutions in the S protein transmembrane domains (TMDs).

S domains	Hydrophilic amino acid substitutions identified	Genotype B		Genotype C	
		HBsAg+ donors (*N* = 353)	OBI donors (*N* = 45)	HBsAg+ donors (*N* = 162)	OBI donors (*N* = 50)
TMD1	Yes	25 (7%)	7 (16%)*	5 (3%)	5 (10%)*
	No	328 (93%)	38 (84%)	157 (97%)	45 (90%)
TMD2	Yes	12 (3%)	10 (22%)**	19 (12%)	9 (18%)
	No	341 (97%)	35 (78%)	143 (88%)	41 (82%)
TMD3	Yes	14 (4%)	20 (44%)**	5 (3%)	18 (36%)**
	No	339 (96%)	25 (56%)	157 (97%)	32 (64%)
TMD4	Yes	41 (12%)	12 (27%)**	28 (17%)	10 (20%)
	No	312 (88%)	33 (73%)	134 (83%)	40 (80%)
Total	Yes	90 (25%)	30 (67%)**	37 (23%)	31 (62%)**
	No	263 (75%)	15 (33%)	125 (77%)	19 (38%)

* *P* < 0.05; ** *P* < 0.01.

### Functional analysis of introducing or removing N-glycosylation sites within MHR

**Figure 2.**
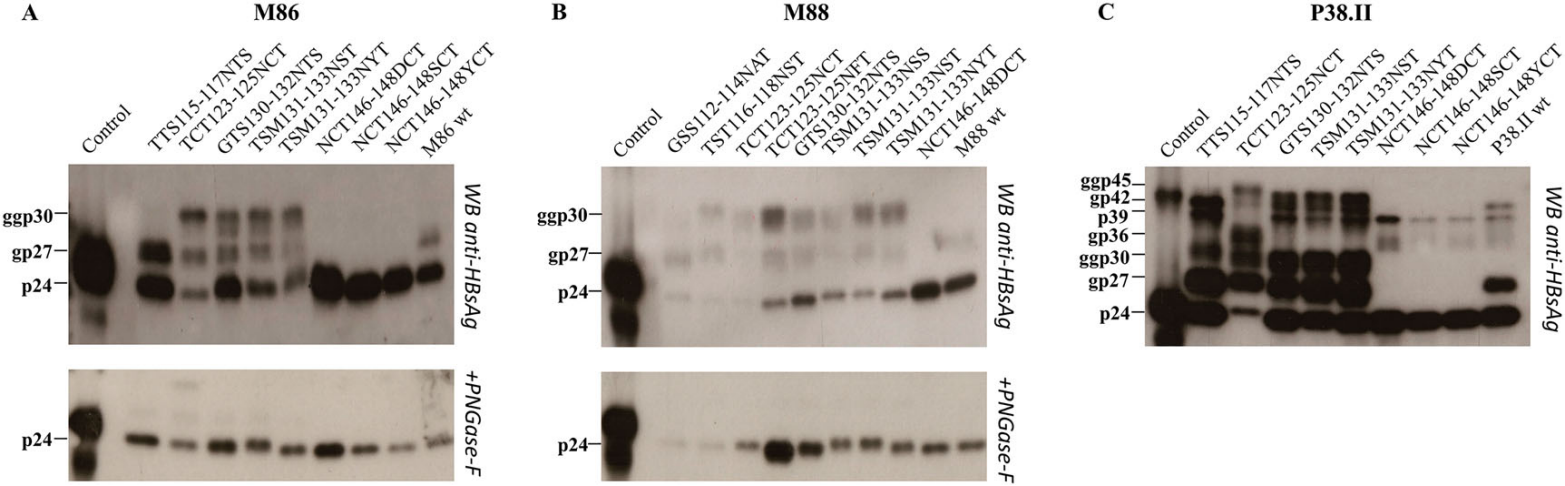
N-glycosylation patterns of mutants and wild-type HBV envelope proteins. HBV envelope proteins produced in transiently transfected HuH-7 cells were analyzed by western blotting using a rabbit R247 antibody targeting the S cytosolic loop. The glycosylated (gp) and nonglycosylated (p) forms of mutated recombinant proteins derived from plasmids M86 (A), M88 (B), and P38.II (C) are indicated. PNGase-F treatment results were also indicated.

Substitutions abolishing the N-glycosylation site at position 146 or introducing additional sites within MHR of OBI_B_ and/or OBI_C_ were selected for functional analysis (OBI_B_: GSS112-114NAT, TST116-118NST, TCT123-125NFT, and TSM131-133NSS; OBI_C_: TTS115-117NTS, and NCT146-148SCT/YCT; OBI_B_ and OBI_C_: TCT123-125NCT, GTS130-132NTS, TSM131-133NST/NYT, and NCT146-148DCT). These mutations were introduced in the S sequence of recombinant plasmids matched for genotypes. N–glycosylation removal or addition was identified by western blot analysis of HBV envelop proteins produced in the culture supernatants of transiently transfected cells. The S- and L-HBsAg proteins were detected in their non-glycosytated (24 and 39 kDa) and glycosylated (27 and 42 kDa) forms in cell cultures transfected with control wt plasmids (Figure 2). Mutants N146D/S/Y showed an expected non-glycosylated profile. A wt glycosylation pattern was observed for mutants GSS112-114NAT in M88 and TTS115-117NTS in M86. All other mutants tested expressed a third S-HBsAg form of 30 kDa, and an additional L-HBsAg protein of 45 kDa when using plasmid P38.II. N-glycosylation appeared to be particularly efficient at position 123, as mutated S protein appeared mainly under the glycol-form bearing two N-linked glycans. Interestingly, mutant TTS115-117NTS in P38.II showed an extra N-glycosylation site in the S but not in the L protein. These data suggested that all sites might be not equally accessible to N-glycosylation because of allosteric differences between the S variants. PGNase treatment verified these data by successfully removing glycan residues.

**Figure 3.**
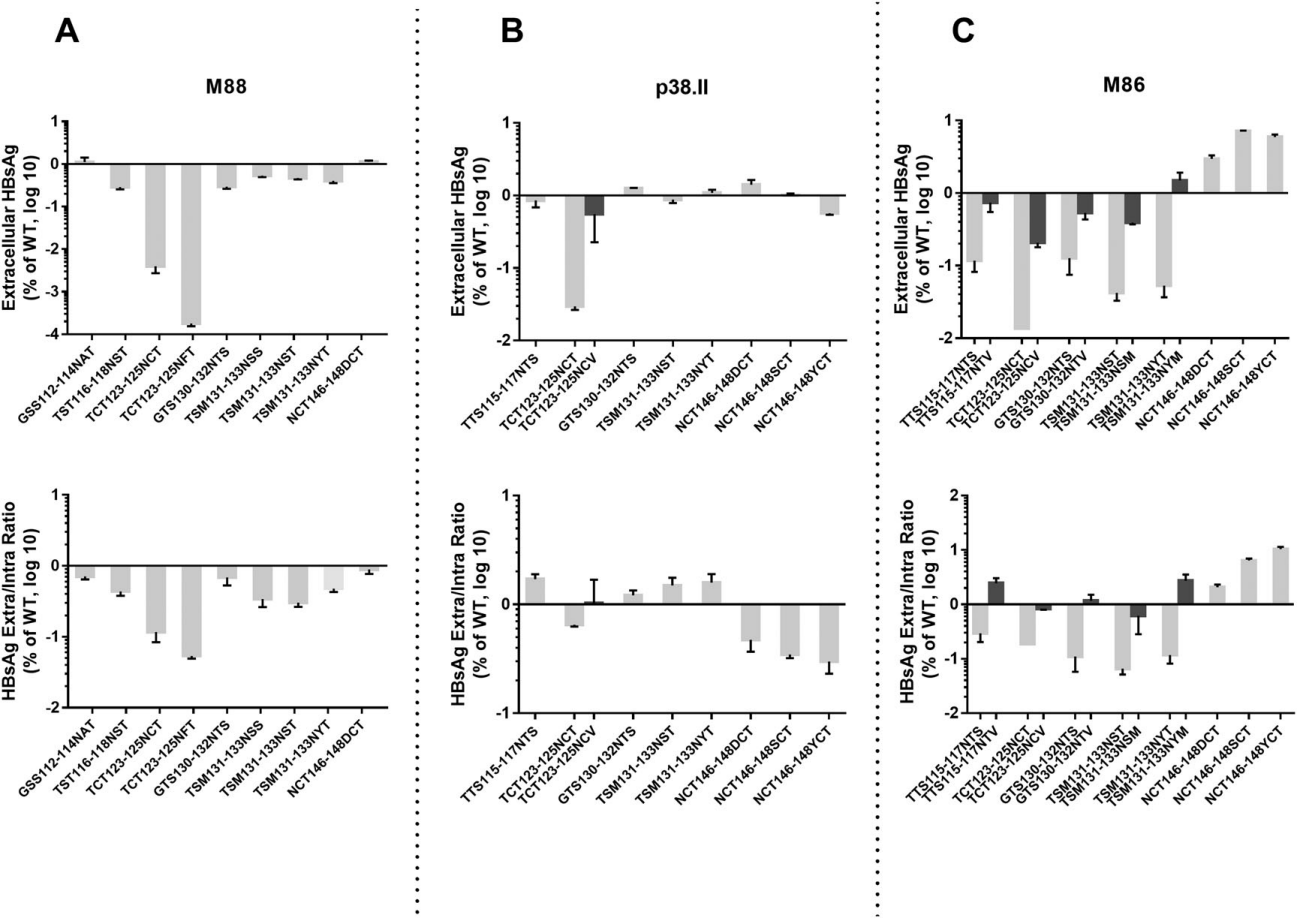
Impact of N-glycosylation mutations in the MHR on intra- and extra-cellular production of recombinant HBsAg in transfected HuH-7 cells. HBsAg levels in the culture supernatants and transfected cell lysates were measured by EIA and normalized according to SEAP activity. The mean relative levels of EC HBsAg production (upper part) and EC/IC ratio (lower part) of mutant recombinant HBsAg were expressed as a log value proportionally to the levels obtained of wild-type HBsAg. Standard deviations are shown for three or more independent cultures. Mutations investigated are indicated and dark bars represent HBsAg production from non-existing mutants created in this study to confirm the impact of additional N-glycans.

To determine the effect of N-glycosylation mutations on subviral particle secretion, HBsAg was measured in both cell lysate and culture supernatant and normalized according to SEAP activity. The average normalized total amount of EC and IC HBsAg produced were similar in the wt control and mutant cultures, except when position 123 was glycosylated (data not shown). Introduction of the N146D/S/Y mutations in plasmid M88 and P38.II by SDM did not significantly alter the EC and IC HBsAg production pattern, although a slight reduction in HBsAg secretion was observed for P38.II mutants, as reflected by the decreased HBsAg EC/IC ratio compared to that of wt HBsAg (5–8 versus 17) (Figure 3). To further investigate the effect of N-linked glycosylation mutations on the HBsAg production pattern, mutations were introduced in plasmid M86 that has been previously associated with an impaired HBsAg secretion pattern [10]. The lack of N-linked glycan at position 146 in M86 recombinant HBsAg resulted in a significant increase in the HBsAg EC/IC ratio, which partially rescued the impaired wt HBsAg secretion (0.11–0.55 versus 0.05). Additional N-glycosylation at position 130 in M88 and P38.II did not substantially change the HBsAg production pattern. Mutations at positions 115, 116 and 131 decreased HBsAg EC production and secretion slightly in M88 (<1 log) and significantly in M86 (>1 log) compared to the corresponding wt HBsAg. In contrast, mutations at positions 115 and 131 resulted in increased secretion of HBsAg in P38.II. Mutations TCT123-125NCT and TCT123-125NFT severely reduced the production of both EC and IC HBsAg (>1 log reduction) of all recombinant HBsAg tested. However, western blotting data showed a similar total amount of S protein forms between mutants carrying a 123 mutation and the other mutants or wt controls by using a polyclonal antibody directed to the S cytosolic domain for detection (Figure 2). Furthermore, the S form carrying two N-linked glycans appeared to be the major glycoform in mutant TCT123-125NCT (Figure 2). This suggested that an additional N-glycan at position 123 might impair EIA detection rather than HBsAg production. Similar results obtained with the double mutant TCT123-125NFT supported this hypothesis, as substitution of cysteine 124 has been reported to negatively affect antigenic reactivity [15].

Double mutants TTS115-117NTV, TCT123-125NCV, GTS130-132NTV, TSM131-133NSM, and TSM131-133NYM were created to verify that the modifications of the HBsAg production pattern observed in the N mutants studied were directly related to the presence of additional glycans and not to the introduction of an asparagine residue in the S protein sequence at critical positions. In the absence of additional glycosylation, these mutants showed a HBsAg production pattern similar to that in the wild-type controls (Figure 3).

### Hydrophilic substitutions in HBsAg transmembrane domains

**Figure 4.**
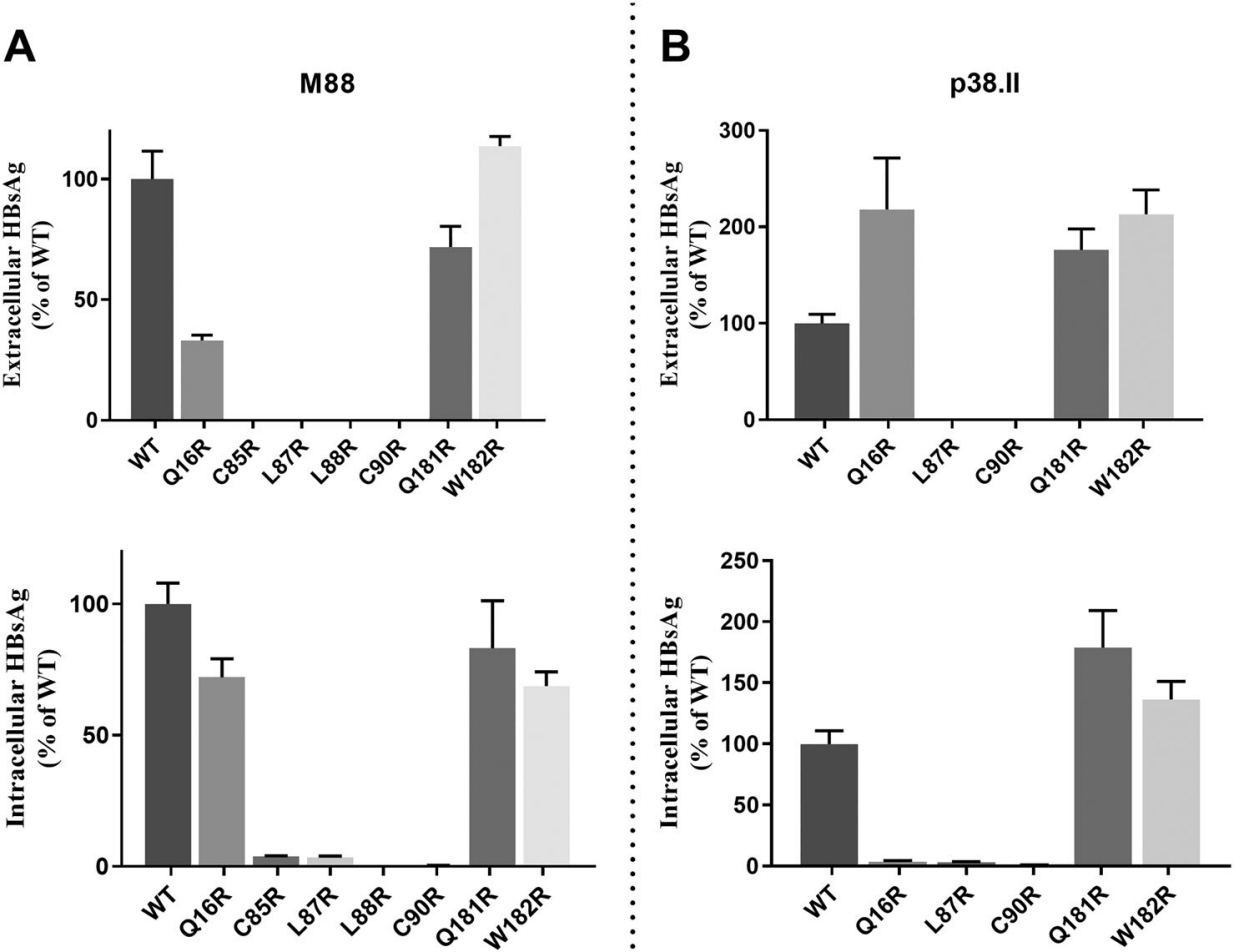
Impact of arginine residue substitutions within the S protein transmembrane domains. Extracellular and intracellular productions of mutated HBsAg were expressed as relative values of wild-type HBsAg production. The mean and standard deviations of at least three independent cultures are shown.

Seven OBI-specific substitutions introducing an arginine residue in S TMDs were investigated: Q16R in TMD1, C85R, L87R, L88R, and C90R in TMD2, and Q181R and W182R in TMD3. Mutations Q16R, Q181R and W182R did not substantially modify the total HBsAg production pattern in M88 and P38.II plasmids (Figure 4). However, mutation Q16R resulted in decreased HBsAg secretion compared to wt M88 (EC/IC ratio: 0.57 versus 1.2) whereas a significant increase in HBsAg secretion was observed in P38.II (EC/IC ratio: 31 versus 0.5). When introduced in M88 and P38.II, mutations C85R, L87R, L88R, and C90R significantly impaired both EC and IC HBsAg production irrespective of the sequence backbone. Particularly, no HBsAg was detected in the supernatants of cultures transfected with mutants L87R and C90R. Western blot analysis of transfected cell lysates verified the absence of detectable levels of intra-cellular S protein carrying L87R and C90R mutations (data not shown).

## Discussion

Among the 1,261 blood donors initially screened as HBsAg-/HBV DNA+ in 29 blood centres across China, 918 donors (72.8%) were confirmed as OBI carriers. These OBI donors shared similar male dominance, normal ALT levels, low viral loads, and ages of ≥40 years compared to OBI blood donors from Northern and Eastern Central China and other Asian countries (Table 1) [7,21,22]. HBV genotype C appeared to be slightly over-represented among OBI donors compared to the randomly selected HBsAg+ donor group as suggested by others [8,21–23]. Irrespective of the genotype, 30.0% of OBI donors were reactive for anti-HBs as previously reported in Chinese OBI donors [8,21–23]. Interestingly, anti-HBs was the only detectable serologic marker in 6.3% of OBIs. This intriguing serologic profile has been reported in 4% of OBIs worldwide but appeared to be more frequent in South East Asia where the profile was observed in 11% of OBI donors [2,7]. The present data confirmed the association of this anti-HBs-only profile with younger age of OBI carriers [7].

OBI sequences were compared with a relatively large number of genotype-matched sequences obtained from HBsAg+ asymptomatic blood donors of the same geographical origins to ensure the robustness of the comparison [7]. The results confirmed the high mutation rate in OBI strains compared to in non-OBI strains and the absence of hotspots of mutations shared by most OBI strains [5,7,8,15]. Nevertheless, two groups of mutations that affected critical structural elements of the S protein were found in a minority of OBIs but at a significantly higher frequency compared to in non-OBI controls (Figure 1 and Table 3). The first group included mutations that modified the N-linked glycosylation pattern within the MHR of S protein either by removing the highly conserved glycosylation site at N146 or by introducing additional glycosylation sites. Functional in vitro analysis confirmed that removal of the N146 glycosylation site was permissive to S protein synthesis and secretion of HBsAg/SVPs when introduced in M88, and partially restored HBsAg secretion in M86 (Figure 3A and C) [16]. The mechanism by which N146 mutants limited the previously documented, but still molecularly uncharacterized, HBsAg secretion deficit in wt M86 remains elusive [10]. In contrast, N146 mutations appeared to slightly reduce HBsAg secretion in P38.II which produced both HBsAg and virions (Figure 3B). This agrees with the results of previous reports indicating that N146 mutation is detrimental to HBV virion production [16].

Mutations TST116-118NST, TCT123-125NCT/NFT, GTS130-132NTS, and TSM131-133NSS/NST/NYT resulted in effective N-glycosylation of the S protein in vitro (Figure 2). In contrast, mutations GSS112-114NAT introduced in M88 and TTS115-117NTS in M86 did not lead to N-glycosylation while TST116-118NST in M88 was glycosylated. This suggested that the position of the NXT/S site, aa environment, or secondary/tertiary structure negatively affects the efficiency of glycosylation or stability of the resulting glycoprotein. Mutations T116N, T123N, G130N, and T131N + M133 T have been reported to reduce antigenicity and immunogenicity and rescue virion secretion in N146 mutants [17–19,24,25]. In the present study, N-glycosylation HBsAg mutants, except for the TCT123-125NCT single mutant and TCT123-125NFT double mutant, showed no significant change in antigenicity. In contrast, mutants TCT123-125NCT and TCT123-125NFT showed a low reactivity in EIA and impaired secretion confirming the critical role of the aa120–124 domain for HBsAg antigenicity and secretion [26]. The extremely low EIA reactivity observed for mutant TCT123-125NFT may be related to the combined effect of creation of an N-glycosylation site by T123N substitution and disruption of a putative disulfide bridge by the C124F substitution [15,25]. Heterogeneous results were obtained when examining the effect on in vitro HBsAg production pattern of additional N-glycosylation in the aa130–aa133 region [19,24]. Consistently decreased HBsAg secretion was observed for the TSM131-133NST/NSS mutants in M86 and M88, while mutants GTS130-132NTS and TSM131-133NST/NYT were found to be associated with an aggravated HBsAg secretion defect in M86 but showed no effect in M88 and a slightly increased secretion in P38.II. These discrepant data suggested that the functional properties of these additional N-linked glycosylation sites depend on a combination of multiple substitutions in the amino acid sequence and likely the secondary/tertiary structure of the protein. N-glycosylation was reported to adversely affect the secretion of HBsAg and virions [16]. Glycosylation mutants may have different effects on the individual secretion pathways used by virions (endosomal sorting complex for transport/multivesicular bodies machinery) and HBsAg (constitutive secretory pathway) [26]. There are limitations to the present study. First, N-glycosylation mutations were studied only in recombinant S protein from non-OBI controls, and data could not be verified in the original OBI strains. Second, analysis of virion secretion is missing and further investigations are needed. Previous studies showed that mutations within the four putative transmembrane alpha-helix domains TMD1-4 of the S protein can impair both HBsAg and virions secretion [10,27–29]. The Q16R substitution in the central part of TMD1 in M88 did not significantly affect the total amount of HBsAg produced but slightly decreased its secretion (Figure 4). In contrast, Q16R appeared to enhance HBsAg secretion by reducing the IC level of HBsAg when introduced in P38.II. Similarly, a previous study reported that the insertion of one amino acid in the centre of the TMD1 alpha-helix blocked HBsAg release, which was partially complemented by the pre-S2 domain, indicating TMD1 not required for HBV envelope protein assembly as a transmembrane domain [13,28]. The separate introduction of substitutions C85R, L87R, L88R, and C90R in the TMD2 alpha-helix significantly impaired both HBsAg production and secretion (Figure 4). Introduction of a positively charged arginine residue in this integral hydrophobic transmembrane domain may alter protein folding and insertion into the ER membrane leading to HBsAg retention and rapid degradation [29]. This agrees with the extremely low or undetectable EC and IC HBsAg levels measured by EIA and western blotting. Moreover, these mutations may affect the intermolecular interactions between the TMD2 domains of S proteins which are essential for the oligomerization of S proteins during HBsAg morphogenesis [13]. The substitutions Q181R and W182R had no significant impact on HBsAg production pattern, suggesting that TMD3 is not essential for S protein oligomerization and HBsAg formation [13,30]. However, in a previous study the OBI-associated P178R substitution prevented HBsAg secretion in two distinct OBI clones and in M88 [10]. It was hypothesized that the presence of a charged arginine residue in TMD3 altered the topology of the HBsAg C-terminal domain. The present data do not support this hypothesis and raise some questions regarding the robustness of the models used to predict the hydrophobic C-terminal domains structure.

In conclusion, occult HBV genotype B and genotype C strains from Chinese blood donors showed higher amino acid variability in their S protein compared to strains from HBsAg+ donors. Two types of mutations in the S region were significantly associated with occult HBV carriage: (i) mutations creating new N-linked glycosylation sites at positions s116, s123, s130, and s131 + s133 or removing the existing site at position s146; and (ii) mutations C85R, L87R, L88R, and C90R within the hydrophobic alpha-helix of transmembrane domain TMD2. Preliminary data suggest that these mutations were associated with reduced antigenicity, various levels of impaired HBsAg secretion, or severe defective HBsAg production and may contribute to the multifactorial occurrence of OBI.
